# Voluntary Behavior and Training Conditions Modulate *in vivo* Extracellular Glucose and Lactate in the Mouse Primary Motor Cortex

**DOI:** 10.3389/fnins.2021.732242

**Published:** 2022-01-04

**Authors:** Alexandria Béland-Millar, Claude Messier

**Affiliations:** School of Psychology, University of Ottawa, Ottawa, ON, Canada

**Keywords:** primary motor cortex, running wheel, biosensor, glucose, lactate

## Abstract

Learning or performing new behaviors requires significant neuronal signaling and is metabolically demanding. The metabolic cost of performing a behavior is mitigated by exposure and practice which result in diminished signaling and metabolic requirements. We examined the impact of novel and habituated wheel running, as well as effortful behaviors on the modulation of extracellular glucose and lactate using biosensors inserted in the primary motor cortex of mice. We found that motor behaviors produce increases in extracellular lactate and decreases in extracellular glucose in the primary motor cortex. These effects were modulated by experience, novelty and intensity of the behavior. The increase in extracellular lactate appears to be strongly associated with novelty of a behavior as well as the difficulty of performing a behavior. Our observations are consistent with the view that a main function of aerobic glycolysis is not to fuel the current neuronal activity but to sustain new bio-infrastructure as learning changes neural networks, chiefly through the shuttling of glucose derived carbons into the pentose phosphate pathway for the biosynthesis of nucleotides.

## Introduction

Glucose is generally seen as the primary, if not sole, metabolic fuel for the brain (Berg et al., 2002). The importance of glucose in the central nervous system is supported by the cognitive deficits and loss of consciousness that accompany hypoglycemia and reduced glucose transport to the brain (Abdul Muneer et al., 2011; Mergenthaler et al., 2013; Patching, 2017), as seen in the GLUT1 deficiency syndrome: a genetic disorder characterized by a deficiency in the facilitative glucose transporter 1 (GLUT1) responsible for shuttling glucose across the blood-brain barrier (Heilig et al., 2003; Marin-Valencia et al., 2012; Yu et al., 2020). GLUT1 deficiency syndrome can be debilitating, but is rarely lethal (Di Vito et al., 2018). The early introduction of a ketogenic diet can prevent epilepsy and many of the deficits associated with GLUT1 deficiency syndrome (Klepper et al., 2004; Friedman et al., 2006; Brockmann, 2009; Klepper, 2012; Klepper and Leiendecker, 2013; Kass et al., 2016). These clinical effects indicate that the brain is capable of retaining its ability to use alternative fuels such as ketone bodies, and that it is not limited to the use of glucose to meet its high energetic needs.

The use of alternative energy sources and pathways for the production of cerebral energy has led to the controversial topic of lactate as a metabolic substrate for energy production. Briefly, the debate centers around the directional flux of lactate, reflecting its role either as a by-product or an energy source. The astrocyte-to-neuron lactate shuttle (ANLS) hypothesis stipulates that the sodium-dependent astrocytic uptake of surrounding glutamate following neuronal activation results in elevated glycolysis within astrocytes, as a response to the energy requirements of the astrocytic sodium-potassium ion pump. This elevated glycolysis leads to an accumulation of lactate, which is then shuttled to surrounding neurons and utilized within the oxidative phosphorylation pathway (selected reference Pellerin and Magistretti, 1994; Magistretti, 2006; Pellerin and Magistretti, 2012). The most direct evidence for this hypothesis comes from *in vitro* studies. On the other hand, the neuron-to-astrocyte lactate shuttle (NALS) hypothesis stipulates that increased neuronal activity upregulates glycolysis within the neurons, resulting in elevated intracellular lactate as a downstream product. This lactate is then shuttled to the extracellular space to be taken up and exported from the central nervous system via astrocytes and their intimate connection with the surrounding vasculature. Though this theory is more in line with the glucose-centric view of brain energetics, evidence for this hypothesis mostly relies on modeling approaches (selected references Simpson et al., 2007; Díaz-García et al., 2017; Dienel, 2017). A full review of the merits and pitfalls of each hypothesis is outside the scope of this work and have been extensively reviewed elsewhere (Jolivet et al., 2010; Mangia et al., 2011; Dienel, 2012; Mason, 2017; Magistretti and Allaman, 2018).

### Meeting the Metabolic Needs of the Brain

The brains energetics needs are met primarily through the function and collaboration of the glycolytic and oxidative phosphorylation (OXPHOS) pathways. For the sake of brevity, we will consider both the tricarboxylic acid (TCA) cycle and mitochondrial respiration (electron transport chain) when discussing OXPHOS. OXPHOS is the primary ATP producing pathway and is considered to occur mostly in neurons (Magistretti and Allaman, 2015; Dienel, 2018) as the majority of cerebral energy goes toward maintaining Na+/K+ membrane potential following signaling (Berg et al., 2002). Glycolysis, on the other hand, produces fewer ATP molecules but has the capacity to occur at a much faster rate (Bui and Thompson, 2006) and feeds the OXPHOS pathway with necessary intermediates, such a pyruvate.

We can consider the possibility that these intermediates are exclusively produced internally if the upregulation of glycolysis within neurons following activation is equivalent to, or surpasses, the demand imposed by OXPHOS. This view is supported by work conducted by Hall et al. (2012) and Díaz-García et al. (2017). Alternatively, we can consider that these intermediates may be provided by surrounding cells, in concert with neurons. Many brain cells, such as astrocytes (Dienel and Hertz, 2001; Bolaños et al., 2010), endothelial cells (Culic et al., 1997; Eelen et al., 2018; Yetkin-Arik et al., 2019), and activated microglial (Ghosh et al., 2018) are considered to be primarily glycolytic, or even provide storage in the form of glycogen, such as in astrocytes. Therefore, these cells could reasonably be expected to produce glycolytic products in excess of any OXPHOS needs, and as such, provide a portion of the required substrates for neuronal OXPHOS.

This cooperative organization and distribution of the hypothesized metabolic specialty across neurons and astrocytes resembles the muscular lactate shuttle proposed by Brooks (1985, 1998, 2009) in which fast-glycolytic-muscles supply slow-oxidative muscles with lactate. Of note, both the ANLS and the shuttle proposed by Brooks are developed to explain energy consumption and exchange in conditions of activation and not resting state. These phenomena are therefore expected to be observed during increased neuronal activity within the brain, as a compensatory and homeostatic mechanism countering the large energetic demand of re-polarizing membranes (Wang et al., 2017) and re-supplying of neurotransmitter vesicles in neurons (Rangaraju et al., 2014). Thus, in response to neuronal activity, OXPHOS and glycolysis are upregulated in both neurons and astrocytes (Ivanov et al., 2014), in addition to increased blood flow required to support enhanced rates of metabolism (Mintun et al., 2001; Chaigneau et al., 2007; Iadecola, 2017). Though both pathways are upregulated in both neurons and astrocytes following stimulation, OXPHOS appears to be preferentially upregulated in neurons whereas glycolysis appears to be upregulated globally within many cell types (Dienel, 2018). There remains controversy as to whether the upregulation of glycolysis occurs primarily in astrocytes or neurons. As this debate resides outside the scope of this article, we refer the readers to other papers (Magistretti and Allaman, 2015; Díaz-García et al., 2017).

### Metabolite and Metabolism Fluctuations Following Activation

Fox and Raichle (subsequently confirmed by various researchers) first brought to light the local de-coupling between glucose and oxygen following neuronal activation (Fox and Raichle, 1986; Paulson et al., 2010; Watts et al., 2018). These extracellular measures completely disrupted our understanding of metabolism. Their observation of a fall in the stochiometric ratio between glucose and oxygen consumption during activation points to an important rise in non-oxidative glucose consumption. In other words, during activation, not all glucose entering the brain is fated to undergo OXPHOS, which begs the question: where does this additional glucose go? The non-oxidative consumption of glucose has three likely fates: (1) efflux out of the central nervous system in the form of lactate as a glycolytic end-product, resulting from an increase in glycolysis that surpasses the immediate capacity for OXPHOS, (2) entering the pentose phosphate pathway for antioxidant and nucleotide production or (3) glycogenesis, the non-oxygen requiring glycogen synthesis pathway.

One of the many tools used to elucidate the flux and fate of metabolites within the brain is the observation of extracellular fluctuation. A robust observation that has emerged from this research is the extracellular decrease in glucose and increase in lactate following and during neuronal activation. These findings have been replicated in different models and under varying conditions (Madsen et al., 1995; Frahm et al., 1996; McNay and Sherwin, 2004; McNay et al., 2006; Kiyatkin and Lenoir, 2012; Sampol et al., 2013; Béland-Millar and Messier, 2018). However, debate remains as to the particular origin of influx and efflux of these metabolites.

The homeostatic metabolic responses engaged following activation (neurotransmitter release), that is to say increased blood flow, decoupling of glucose-oxygen use increased OXPHOS and glycolysis, somehow result in increased extracellular lactate and decreased extracellular glucose. Interestingly, however, is that this trend in extracellular metabolite fluctuation is highly susceptible to training/practice and novelty. Habituation, in the form of repeated stimuli, such as in the novel object task (Bauch et al., 2017; Béland-Millar and Messier, 2018), or repeated behavior, such as wheel running (Grill-Spector et al., 2006; Fisher et al., 2016), has been shown to decrease both the signaling and consequently the metabolic requirements of the activated cortical area(s). Historically this has been attributed to the development and increased efficiency of the neural networks.

### From the Where to the What of Energy Metabolism?

The ANLS and NALS hypotheses focus and differ primarily on *where* glucose and lactate are produced and consumed, with little regard to the function they serve beyond energy production. A complementary hypothesis has recently emerged, focusing on the function of these various products and metabolic pathways, suggesting diverging functions for energy derived from aerobic glycolysis (glycolysis terminating in lactate despite sufficient oxygen levels) and OXPHOS. This hypothesis proposes that much of the energy derived from aerobic glycolysis is primarily used to sustain the energy requirements for the physical building of neurites and dendritic spines accompanying new learning (Fellows et al., 1993; Bauernfeind et al., 2014; Goyal et al., 2014; Shannon et al., 2016; Zhou et al., 2019). This hypothesis is consistent with the observations of decreased extracellular lactate (Takita et al., 1992; Béland-Millar and Messier, 2018), signaling (Grill-Spector et al., 2006; Fisher et al., 2016) and altered metabolism (Toga and Collins, 1981; Gonzalez-Lima et al., 1989) following habituation.

The studies herein were conducted to validate a motor task in the study of brain metabolism. In the present experiment, we examined the effect of short- and long-term wheel-running training, as well as climbing and spinning, on changes in extracellular glucose and lactate using micro biosensors inserted in the primary motor cortex. We aimed to confirm if the tasks result in the expected metabolite fluctuations in other behavioral tasks and stimulation. Validating these new behavioral tasks was important to the authors as many common behavioral assessments are incompatible with tethered systems that measure metabolite fluctuations. In addition, many tasks rely on the assumption that the rodents are engaging with their environment and learning but these behaviors are limited in their ability to be observed and measurable. Motor behaviors that would elicit similar and robust responses are easier to assess, either in terms or speed or distance, as well as easier to determine when the mice are or are not engaging in said behavior.

## Experimental Procedures

### Animals

Thirty-six, 14- to 16-week old, male CD1 mice (Charles River Canada, St-Constant, QC, Canada) were individually housed and maintained on a reverse 12-h night/day cycle with lights on at 7 p.m. Mice had *ad libitum* access to standard chow (Teklad Global 18% Protein 2018, Teklad Lab Animal Diets, Envigo, Mississauga, Canada) and water, unless otherwise specified. All testing was conducted during the night phase of the cycle with the use of dim red lighting. All procedures in this study followed the Canadian Council on Animal Care guidelines and were approved by the University of Ottawa Animal Care Committee.

### Surgery

Extracellular glucose and lactate measures were obtained with the use of biosensors inserted into the primary motor cortex. The surgical procedures used are identical to those detailed in previous work (Newman et al., 2011; Béland-Millar et al., 2017; Béland-Millar and Messier, 2018; Scavuzzo et al., 2020). Briefly, under anesthesia (Isoflurane, Fresenius Kabi Canada Ltd., Richmond Hill, ON, Canada) and analgesia (0.05 mg/kg subcutaneous buprenorphine hydrochloride, Reckitt Benckiser Healthcare, Hull, North Humberside, United Kingdom), a guide cannula (Pinnacle Technology, KS, United States) was placed above the right and left primary motor cortex (M1; from Bregma, ±1.8 mm lateral and +1.1 mm anterior) just below the skulls surface, according to the stereotaxic atlas of Franklin and Paxinos (Paxinos and Franklin, 2008). The cannulas were held in place with the use of 0.10-inch screws (Pinnacle Technology, KS, United States) and a UV-polymerized compound (UV Clear Fly Finish, Ashland, OR, United States).

### Testing Procedures

**FIGURE 1:**
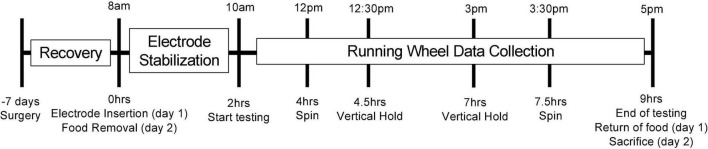
Illustration of the testing timeline. This figure illustrates the testing timeline. The mice were acclimated to the facility for 3 weeks prior to testing. Seven days prior to testing, the mice underwent surgery for cannula implantation. On the morning of the testing (day 1), the mice were lightly anesthetized, and the electrodes were inserted following calibration. The mice were then placed into their testing cages with access to water, bedding and a running wheel. The electrodes were left to stabilize for 2 h while the mice acclimated to their testing cage and familiarized themselves with the running wheel. Following this acclimation and stabilization period, spontaneous and voluntary running events were recorded for the next 7 h. Twice during this testing window, the mice were also subjected to our effortful behaviors (spin and vertical hold) in a counterbalanced order. Once the testing period concluded, food was returned to the cage. Once the following day (day 2), testing procedures were identical with the exception of food removal in the morning (replacing the electrode insertion), and calibration of the electrodes at the end of the day, after which the mice were euthanized. The group of mice receiving access to a running wheel 4 weeks prior to testing is the only variation to this timeline. Surgery for this group of mice still occurred 1 week prior to testing (3 weeks into their exposure to the running wheel).

After a week of recovery, two electrochemical electrodes (7004-Glucose-C and 7004-Lactate-C; Pinnacle Technology Inc., Lawrence, KS, United States) were inserted into the guide cannula so that the 1 mm sensing cavity of the electrode protruded from the guide cannula and rested in the primary motor cortex. The characteristics of these commercially available electrodes have been presented previously (Wilson and Gifford, 2005; Wakabayashi and Kiyatkin, 2012, 2015; Kiyatkin et al., 2013; Béland-Millar et al., 2017). The mice were then placed in their testing cage with water and a free-standing running wheel. The mice were left to acclimate to their testing cage and running wheel for 2 h while the electrodes stabilized. Running events occurring during this 2-h stabilization were not included in the analysis. Otherwise, all running events occurring during the testing period (between 10 am and 5 pm) were include in the analysis. The running wheel was intentionally included during the acclimation so that the mice could habituate and start to learn how to use it. Preliminary experiments showed that initial interactions with the running wheel were clumsy and brief, however the 2-h calibration window was sufficient for the mice to start readily engaging with and using the running wheel for longer periods of time. In addition to these running bouts, the mice were tested for effortful behaviors (vertical hold and spin, see below) twice a day, for 2 days, with 20-min intervals between the behaviors and in a counterbalanced fashion. The length of the manipulation and interval was established during preliminary experiments. This chosen interval was sufficient for extracellular metabolite levels to return to baseline levels. See Figure 1 for an illustration of the testing timeline.

#### Voluntary Wheel Running

The mice had unlimited access to a running wheel (Low-Profile Wireless Running Wheel for Mouse, Med Associates, VT, United States) during testing procedures. The number of rotations/minute was recorded by the Running Wheel Manager Data Acquisition Software through a wireless hub (Med Associates Inc., VT, United States) and was used to compare run speed where appropriate.

Because mice were free to use the running wheels at any time, there were differences in running speed and duration within and across mice. In previous projects with the same procedure, we had observed that the amplitude of change in extracellular glucose and lactate in the motor cortex correlated with the length of time the mice ran in a single running bout. These observations and the uneven distribution of running bout lengths within each animal led us to analyze the running bouts across animals as a function duration in order to extract the effects of running bout length on extracellular glucose and lactate. Bout length was measured in seconds, representing the duration in which the mouse engaged in running behavior on the running wheel. Bout speed was measured by dividing the number of wheel rotations by the bout length. We grouped runs based on their duration: 30–60, 60–120,120–180, 180–240, and 240+ seconds. For example, the graph depicting 30-s events includes the running events from all animals that lasted at least 30 s but did not exceed 59 s.

We also evaluated running bouts as a function of experience. Short-term experience was evaluated with 24 h of prior access to the running wheel. These mice underwent the usual surgery and testing schedule. Mice who had long-term exposure to the running wheel were given access to it in their home cages, 4 weeks prior to testing. On the third week of their access, mice underwent surgery and were implanted with the guide cannulas (see above). They then underwent the week of recovery and their 4th week of access to the running wheel. Following the 4th week, they were brought in for testing and the electrodes were inserted. For both of these conditions (short-term and long-term experience), only runs of 240 s or longer were retained and analyzed due to larger and more consistent change in glucose and lactate extracellular levels produced. This allowed a better comparison with the less experienced mice (see section “Results”).

#### Effortful Behaviors

Pilot data showed that the voluntary wheel running induced changes in extracellular glucose and lactate that were smaller in magnitude than those produced by electrical stimulation (Hu and Wilson, 1997a). Moreover, the magnitude of these changes in extracellular metabolite levels were inconsistent, both across and within mice, but appeared to be anecdotally related to the duration and speed of the running event. We therefore hypothesized that the differences we observed with running bouts were proportional to the effort mice exerted while running. We tested this hypothesis by adding what we estimated to be motor tasks that are more challenging than voluntary wheel running, with the prediction that greater extracellular glucose and lactate changes in the motor cortex would be observed. These behaviors were coined “effortful behaviors.” Pilot experimentation showed that the two motor tasks that were compatible with the tethered electrode set-up and elicited strong glucose and lactate changes were the Vertical Hold and Spinning motor tasks described in the next section. These tasks used the propension of mice to re-orient themselves toward the top of a vertical plane and climb toward it. Typical tasks, such as the rotarod, could not be utilized with this tethered experimental setup as the fall risks pulling the electrodes out.

#### Vertical Hold

In this task, the mouse was placed on a vertical metal mesh (10 × 17 inches) with half-inch square holes. The mesh was held vertically and still as the mouse was free to explore for 2 min. This was initially used a control for the following behavior in which the mesh was moved. As the mice habituated to this task, they tended to orient and move toward the top of the mesh and spend more time there.

#### Spin

The mouse was placed on the same mesh as previously described but for this task, the mesh is slowly spun around in the vertical plane which led the mice to re-orient themselves toward the top of the mesh. A mouse was initially spun at the rate of 1 full rotation per 4 s. After completing four full rotations, the mouse was then rotated counterclockwise at the same rate. Once another four rotations have been completed, the mouse was once again spun clockwise – however, this time a full rotation is completed every 2.5 s for another four rotations before completing the same conditions counter-clockwise. In summary, the rotations were done in sets of 4; the first two sets being slower, the last two being faster and switching between clockwise and counterclockwise rotations between each set of four rotations. This pattern was repeated for 2.5 min after which the mouse was returned to its cage.

Those particular speeds were chosen following pilot experimentation. At slower speeds, most mice were able to continue their normal pattern of behavior and exploration, seemingly undisturbed by the spinning and reaching the top of the mesh without issue. At higher speeds, most of the mice fell off of the mesh, unable to hold on. During the slower testing (4 s per full rotation), mice attempted to re-orient themselves to face the top of the mesh and a few even explored the mesh further, managing to reach the top. However, during the faster spinning (2.5 s per full rotation), the mice continued to attempt to reorient themselves toward the top of the mesh but were often unable to do so effectively. They remained in the middle of the mesh, with little exploration toward the top but did not typically fall.

### Statistical Analysis and Relative Measures

All extracellular data were standardized by dividing the readings with their respective pre-event baseline^1^ and multiplying the result by 100 to obtain relative percentage change from baseline. When necessary, the data was averaged every few seconds. Glucose and lactate were analyzed separately with a between-within (split-plot) mixed ANOVA (α = 0.05), and the data obtained for each injection was compared to a control injection group as well as baseline. Unless otherwise stated, the data met the respective assumptions of the analysis. If sphericity or homogeneity was violated, a Greenhouse–Geisser correction was applied. Due to the *a priori* decision and interest in differences across groups, *post hoc* pairwise comparison tests (α = 0.05) were computed in order to compare the experimental groups to the saline controls as well as compare the experimental groups amongst themselves. All statistical analyses were performed using SPSS v. 23 (IBM).

To facilitate visualization of data, significant differences (*p* < 0.05) from baseline are illustrated directly on the graphs with the use of horizontal lines below the data. Significant differences from baseline for extracellular lactate are indicated via a thick line, whereas significant baseline differences for extracellular glucose are indicated via a thin line. Differences from respective control groups are indicated by a gray shaded rectangle around the respective data points. Where applicable, data typically considered to be “non-significant” (probability greater than 0.05) may still be discussed in order to provide a more complete understanding of the results^2^.

## Results

### Extracellular Lactate Rises and Extracellular Glucose Decreases in the Motor Cortex During Voluntary Wheel Running

**FIGURE 2:**
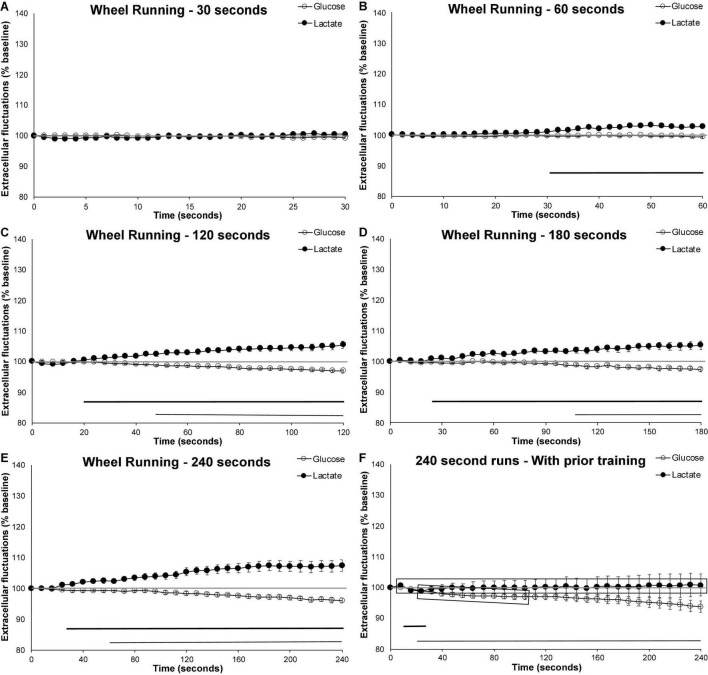
Extracellular glucose and lactate fluctuations during voluntary wheel running. Extracellular cortical glucose (open circle) or lactate (filled circle) during voluntary wheel running for individual running events lasting between **(A)** 30–59 s (*n* events = 32, 10 mice), **(B)** 60–119 s (*n* event = 58, 10 mice), **(C)** 120-179 s (*n* events = 35, 10 mice), **(D)** 180–239 s (*n* events = 31, 10 mice), or **(E)** 240 + seconds (*n* events = 36, 10 mice). **(F)** Running events of at least 240 s taken from mice (*n* = 4) who were previously exposed to the running wheel for 4 weeks (*n* events = 29). Time zero corresponds to the mouse initiating running. Values are expressed as a percent of their baseline taken prior to activity and the error bars indicate the standard error of the mean. The mid-graph gray line shows baseline (100%) value. Horizontal lines below the data indicates significant (*p* < 0.05) differences from baseline for both extracellular glucose (thin line) and lactate (thick line).

Voluntary wheel running in mice who have not been previously exposed to the running wheel showed different patterns of extracellular metabolite fluctuations based on the length of their run. Wheel running behavior that occurred for at least 2 min (120 s) was associated with increased levels of cortical extracellular lactate as well as decreased levels of extracellular cortical glucose. In these longer runs (120 s or more), the rise in extracellular lactate occurred approximately 20 s following the start of the behavior (Figure 2). Interestingly, this rise in extracellular lactate did not appear to occur in the shortest run duration. In 30 s runs, neither extracellular glucose nor lactate significant varied from baseline. In 60 s runs, only lactate began to increase beyond baseline levels. When compared to the 30-s runs, extracellular lactate rose to significantly higher levels for the 120- (*p* = 0.007), 180- (*p* = 0.002), and 240-sec runs (*p* < 0.001). Despite lactate increasing within 20 s of the start of the running behavior in longer bouts, this increase in lactate was not observed in the 30 s runs, perhaps suggesting some sort of physiological preparation.

Decreases in cortical extracellular glucose from baseline appeared later than the extracellular rise in lactate, significantly dropping below baseline levels no earlier than 50-s or as late as 110-s following the start of the voluntary wheel running, as illustrated in Figure 2. When compared to the 30-s runs, the decrease in extracellular glucose was only significantly different for the 240-s runs (*p* = 0.026). This seems to indicate that compensatory mechanisms are capable of maintaining extracellular glucose levels at a steady state for some time. These mechanisms likely include increases in blood flow and the use of internal glycogen stores to compensate for the heightened metabolic rate.

### Effect of Experience: 4-Week Prior Access to a Running Wheel Attenuates the Rise in Extracellular Lactate and Hastens the Decrease in Extracellular Glucose

To facilitate the investigation of the effect of training on extracellular metabolite fluctuations, the longer (240 s) bouts of running were used as they most clearly demonstrated the diverging extracellular glucose and lactate patterns. When mice were given free-access to a running wheel 4 weeks prior to testing, the exercise-induced increase in extracellular lactate was attenuated (*p* < 0.001), while the decrease in extracellular glucose occurred earlier (*p* < 0.034) when compared to naïve mice (see Figures 2E,F). Figure 2F shows that the decrease in extracellular glucose occurred approximately 40 s earlier (thin line indicating significant different from baseline), and that this decrease was more pronounced (shaded gray rectangle indicate significant difference form control). Extracellular lactate almost never varied from baseline and was significantly different from the naïve counterpart for the duration of the running behavior.

### Effortful Behaviors Have a Larger Impact on Extracellular Lactate Than Voluntary Wheel Running

**FIGURE 3:**
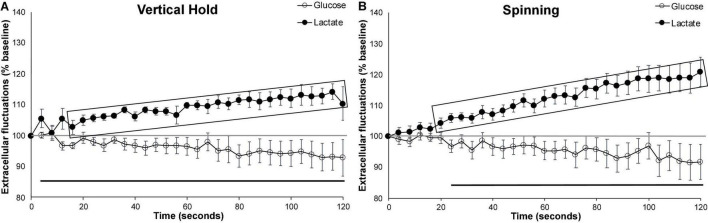
Effortful behaviors. Average extracellular cortical glucose (open circle) or lactate (filled circle) during **(A)** all vertical holds events (*n* = 7), **(B)** all spinning events (*n* = 10). Gray-shaded rectangles surrounding the glucose (open circles) and lactate (filled circles) curves indicate significant differences from respective controls (120 s runs). Extracellular glucose and lactate did not significantly differ between the vertical hold and spinning conditions. Horizontal lines below the data indicates significant (*p* < 0.05) differences from baseline for both extracellular glucose (thin line) and lactate (thick line). The mid-graph gray line shows baseline (100%) value. Gray-shaded rectangles surrounding the glucose and lactate curves indicate significant differences from the running wheel (120 s), for both extracellular glucose (open circle) and lactate (filled circle).

When compared to 2 min (120 s) of wheel running (Figure 2C), both the vertical hold and the spinning conditions (Figure 3) significantly increased extracellular lactate (*p* = 0.007 and *p* < 0.001, respectively), with no significant impact on extracellular glucose. In fact, extracellular lactate during effort behaviors was significantly higher than the levels observed during the 120 s wheel running for almost the entire duration of the testing (shaded rectangles in Figure 3). However, neither extracellular glucose nor lactate significantly differed between the two effortful conditions.

### Effect of Novelty: Gained Ability vs. Habituation

To distinguish between the effect of novelty and improved ability due to prior exposure, we also compared extracellular glucose and lactate on the first and second day of testing for both running wheel (240 s bouts) and spinning. Day 1 of testing represented novel manipulations (spinning) and first access to the running wheel whereas day 2 represented either two prior occurrences of the spinning task or a 24-h prior exposure to the running wheel.

**FIGURE 4:**
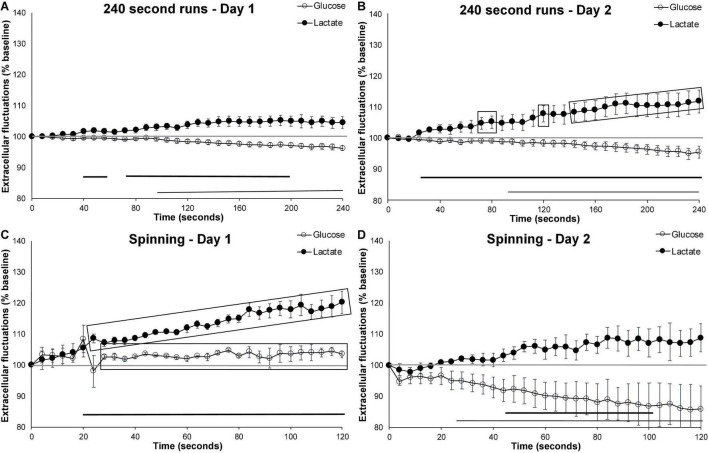
Effect of novelty on extracellular glucose and lactate. Comparing extracellular cortical glucose (open circle) or lactate (filled circle) on day 1 and day 2 of testing. **(A)** Running events of at least 240 s on the first day of running wheel access (*n* events = 23, *n* mice = 10). **(B)** Running events of at least 240 s on the second day of running wheel access (*n* events = 12, *n* mice = 10). **(C)** Spinning events on the first day of testing (*n* = 3) and **(D)** spinning events on the second day (*n* = 4). Gray-shaded rectangles surrounding the glucose and lactate curves indicate significant differences from day 1 and day 2 of testing, for both extracellular glucose (open circle) and lactate (filled circle). Horizontal lines below the data indicates significant (*p* < 0.05) differences from baseline for both extracellular glucose (thin line) and lactate (thick line). The mid-graph gray line shows baseline (100%) value.

When compared to the first day of wheel running, running behavior in mice who were exposed to the running wheel for 24-h trended toward increased extracellular lactate beyond the levels observed on day 1 (*p* = 0.057). This was particularly evident during the second half of the running bout (Figures 4A,B). Comparing the speed (number of wheel rotations/second) of these 240 s runs revealed that mice ran faster on the second day (*p* = 0.012; Day 1, Mean = 0.66, SEM = 0.018; Day 2, Mean = 1.07, SEM = 0.043). This suggests that, between the first and second day of exposure, the mice gained proficiency with the running wheel, and were able to exert more effort. The second day of testing may more accurately reflect normal brain activation due to exercise rather than the novelty/habituation of day 1.

In contrast, comparing the spinning events on the first and second day of testing (Figures 4C,D) revealed an attenuation in extracellular lactate (*p* = 0.017) and a trend toward further decreases in extracellular glucose (*p* = 0.055). These results are similar to those observed following the 4 weeks of habituation to the running wheel which suggests that the mice either quickly habituate to this manipulation or that activation on day 1 is more representative of novelty than motor activation as a result of the behavior in which they are engaged. Altogether, these results suggest that novelty and experience have differential short-term effects on extracellular lactate fluctuation.

In summary, long-term exposure and training with a running wheel attenuates the rise in extracellular lactate while simultaneously hastening the decrease in extracellular glucose. Short-term (24 h) exposure and training with a running wheel increases extracellular lactate: This could likely be the result of an improved ability to use the running wheel, subsequently allowing more effort to be exerted. Lastly, results from the more passive behavior (being spun rather than choosing to run) indicates that the novelty induced increase in extracellular lactate is short lived. This latter observation is corroborated with our previous findings with the novel object task, where fluctuations in the primary visual cortex were quickly attenuated upon a second presentation of the object (Béland-Millar and Messier, 2018).

## Discussion

### Technical Limitations of the Biosensors

When interpreting the current results, it is important to consider the technical limitations of the methodology used. Though extremely sensitive with high temporal resolution, the inserted biosensors measure relative changes in metabolite concentrations within the extracellular space. Techniques, such as microdialysis, can provide more accurate measurements of absolute values, however they lack the temporal resolution opted for in these experiments. Once inserted, the electrodes undergo gradual “bio-fouling” as various biological materials attach themselves to the electrodes. This results in an extremely small, gradual but constant decline in the signal. This signal decreases progressively in hours rather than minutes and do not have a direct impact on our acute extracellular sampling during manipulations, as has been previously demonstrated (Béland-Millar et al., 2017). However, this gradual decline and the slight variations in electrode manufacturing, results in variability of the absolute values measured, both across and within (from one day to another) mice. In order to be able to compare the values both within and across mice, the observed fluctuations are more accurate as measures relative to pre-event baselines. The data was transformed from the raw nA values to relative changes in extracellular fluctuations, as described in the methods, to allow for comparison within and across mice.

**FIGURE 5:**
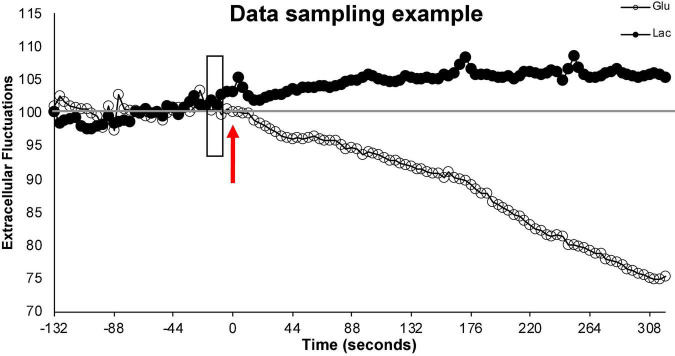
Illustration of data sampling. This figure illustrates a single run and demonstrates the sampling stability of the electrodes prior to behavior. Time 0 and the arrow (red) represents the start of the run. The 2+ minutes of extracellular values (% from baseline) prior to the start of the run (left of the arrow) represent fluctuations in extracellular glucose (open circle) and extracellular lactate (filled circle) during normal (non-running) activity in the testing cage. These values remain close to baseline. The shaded rectangle represents the 10 s of data that is averaged to create the individualized baseline with which the relative extracellular values during this run will be calculated. The extracellular values (% from baseline) to the right the arrow represent the relative extracellular glucose and lactate values during the running behavior. To simplify the representation, data points were averaged every 4 s.

In addition to the slight decline in sensitivity, electrode insertion damages tissues resulting acute and chronic immunological responses (Kozai et al., 2015). In the short term (minutes to hours) electrode insertion may impact regional blood flow through the creation of vascular occlusion or effects on the BBB. Within hours, glial activation is induced which can, over the course of days, encapsulate the electrode. This encapsulation eventually results in the primary measurement of reactive tissue or may cause a slight delay in measuring extracellular metabolites as they cross this additional barrier. These impacts are mediated by insertion of the electrode immediately prior to the testing, rather than at the time of surgery, and by limiting our continuous measurements to 2 days. In lab testing demonstrated that extracellular measurements were robust and replicable across this timeline, but occasionally began to differ on the third day of continuous sampling. Unaveraged data also reveal changes within a second of manipulation or behavior, suggesting that the impacts of bio-fouling on the sampling delay is likely less than the sampling speed of the electrodes (1 s) (Figure 5).

Since the electrodes sample the extracellular space, they measure net metabolite fluctuations in the extracellular space of the motor cortex without providing information on the direction of the flux. Therefore, the glucose and lactate measures obtained with the electrodes reflect the net result of blood-brain barrier (endothelial) transport both in and out of the brain, as well as astrocytic and neuronal influx and efflux of these same metabolites within the brain parenchyma. For example, an increase in extracellular lactate could represent either increased transport of lactate across the blood-brain barrier into the extracellular space, increased neuronal glycolysis resulting in lactate export or lactate shuttling from astrocytes into the extracellular space, among other possibilities. Our strategy in the present experiment and previous ones (Béland-Millar et al., 2017; Béland-Millar and Messier, 2018) has been to measure extracellular glucose and lactate in many different situations together with peripheral metabolic challenges to derive a number of hypotheses on the movement of glucose and lactate under various conditions of brain activity and metabolic status. Our hypotheses also rely on previous experiments that have examined glucose and lactate metabolism either *in vivo* or more often in cell cultures and brain slices preparations.

#### Electrical vs. Motor Stimulation

In the present study and our previous ones, we examined the effect of *in vivo* behavioral or sensory stimulation on brain extracellular glucose and lactate. There is evidence that *in vivo* wheel running is sufficient to induce neuronal activity in the primary motor cortex, and that this neuronal activity fluctuates with the speed of the wheel running (Fisher et al., 2016). Our results confirmed a comparable impact of voluntary wheel running on extracellular metabolite fluctuations as those obtained either by discrete stimulation (Hu and Wilson, 1997b) or alternative behavioral testing (McNay et al., 2006).

Interestingly, if we compare our behavioral results to those of discrete electrical stimulations, such as the work done by Hu and Wilson, we observe a similar pattern. Hu and Wilson measured extracellular glucose, lactate and oxygen in the dentate gyrus of anesthetized rats following performant path stimulation (Hu and Wilson, 1997b). Two patterns of response were observed by Hu and Wilson: an immediate short-term pattern and a longer term one over the course of repeated stimulations. The immediate pattern was a sharp 10% decrease in glucose and oxygen, and a 25–35% decrease in lactate followed by a quick rebound that overshot briefly the pre-stimulation values. The overlaid long-term pattern was a progressive increase in extracellular lactate (up to 200% baseline), a moderate decrease in extracellular glucose (80% of baseline) and a very small (5%) increase in oxygen. When the stimulations ended, glucose and oxygen returned to baseline within 5 min, while lactate was still at 20% above baseline 20 min later. In a previous dataset, we observed that the initiation of any movement was associated with a brief (3 s) 13% decrease in extracellular glucose and a 22% decrease in lactate in the motor cortex, which are comparable in size but much briefer than those observed following electrical stimulation. In the present experiment, sustained running produced a similar de-coupling in extracellular glucose and lactate. However, the increase in lactate and decrease in glucose observed here were much smaller than those observed with electrical stimulation. The results suggest that neuronal activation induces two different processes. The first process, occurring within seconds of stimulation (Hu and Wilson, 1997b) as well as subsequently to motor initiation (prior data set), appears to be a rapid and short-term depletion of glucose and lactate from the extracellular space followed by a longer-term and progressive accumulation of lactate accompanied by a progressive decrease in extracellular glucose that continues as long as the behaviors are occurring. At face value, these results suggest that immediately after brain stimulation, extracellular glucose and oxygen are depleted. This depletion is likely the result of increased oxidative phosphorylation of glucose. As discussed in the introduction, neuronal activation is an energetically costly process, and most energy in the brain is provided through oxidative phosphorylation of glucose. The short-term decrease in extracellular lactate that occurs simultaneously suggests that lactate is also used as metabolic fuel during this early activation either by neurons or other cells. Running, and general motor behavior, would increase circulating lactate, and given that lactate transport is dependent upon a concentration gradient, decreased extracellular suggests lactate is entering brain cells not exiting the brain. However, our previous experiment examining the impact of visual stimulation on lactate and glucose in the visual cortex found a similar lactate increase in the visual cortex. Therefore, this increase is unlikely to be due exclusively to lactate released by muscle activity entering subsequently the brain. Extracellular lactate produced from glycogen breakdown may be a source of metabolic support, enabling appropriate brain function (Suh et al., 2007; Brown and Ransom, 2015; Matsui et al., 2017) but recent evidence suggests that glycogen may support astrocytic, rather than neuronal energy needs, thus sparing substrates (e.g., lactate, glucose) for neuronal use (DiNuzzo et al., 2010; Dienel and Cruz, 2015; Dienel and Rothman, 2020).

The secondary process, represented by the longer-term accumulation of lactate in the extracellular space and the continued decrease in extracellular glucose, suggests elevated glucose consumption. Elevated glucose consumption, accompanied with elevated extracellular lactate, points to a significant portion of the metabolized glucose (or glycogen) being converted to lactate and shuttled out of the cell. Importantly, the type of cell producing this lactate has been extensively debated (Díaz-García and Yellen, 2018; Fernández-Moncada et al., 2018; Yellen, 2018), and it is unclear if this lactate is shuttled out of the cell as waste product or shuttled out of the astrocytes as a potential energy source for neurons.

The hypothesis that OXPHOS and aerobic glycolysis both occur during neuronal stimulation is consistent with the well-described decoupling between glucose utilization and oxygen consumption measured by Positron Emission Tomography during brain activation (Fox and Raichle, 1986). If the increase of extracellular lactate is directly related to neuronal oxidative glycolysis, then the examination of the time course of extracellular glucose decrease and extracellular lactate may provide additional information. Although brain electrical stimulation is a useful tool to examine neuronal activation, it does not reproduce the varied pattern of neuronal activation produced by *in vivo* non-anesthetized sensory stimulation or motor activity. In the next section we examine the temporal pattern of extracellular glucose and lactate produced in the motor cortex by different behaviors.

### Progressive De-Coupling of Extracellular Glucose and Lactate During Motor Behavior

We showed that as wheel running continues, extracellular lactate continues to rise, and glucose continues to decrease. We previously demonstrated that this same de-coupling pattern of occurs in the visual cortex of freely moving mice when visually stimulated (Béland-Millar and Messier, 2018). Our observations are also compatible with the reduction in extracellular glucose (McNay et al., 2000, 2001; McNay and Gold, 2001; Newman et al., 2011) and increase in extracellular lactate (McNay et al., 2006; Newman et al., 2011) levels in the hippocampus during spatial working memory testing.

As we have found, the magnitude of the decrease appears to be proportional to the cognitive demands of the tasks (McNay et al., 2000, 2001; McNay and Gold, 2001). Interestingly, others have found that the decrease in extracellular glucose during cognitive testing is magnified in older animals and is accompanied by impaired performance (McNay and Gold, 2001). However, the fact that this performance impairment can be alleviated by an ip or intra-cranial injection of glucose, suggests that the large decrease in extracellular glucose in older animals may be due to a mismatch between the neuronal availability and demand of glucose. Together these results support the importance of glucose availability and is consistent with the observation that new training produces an increase in GLUT1 glucose transporter expression in the hippocampus for memory training (Choeiri et al., 2005) and in the sensorimotor, motor cortex and cerebellum in mice given 48-h access to a running wheel (Allen and Messier, 2013). In that last experiment, running wheel access also increased the expression of beta-tubulin III, a neuron-specific microtubule, which suggested that the increased GLUT1 expression was also associated with neuronal remodeling (Allen and Messier, 2013).

A second observation is the temporal de-coupling between lactate and glucose. When the animal starts running, the decrease in extracellular glucose appears to lag from 10 to 90 s behind the increase in extracellular lactate. We propose that this delay represents the cells initial reliance on astrocytic glycogen stores to meet energetic demands following activation (Brunet et al., 2010). This buffer provided by the internal glycogen stores limits the need for cells to use intracellular glucose pools (Dienel, 2018), sparing extracellular glucose. Subsequently, when the initial local supply of glycogen is reduced, intracellular glucose pools are more heavily relied upon, thereby reducing extracellular glucose. The increased reliance on astrocytic glycogen may result in elevated extracellular lactate, as suggested by Dienel (2019). There is, however, additional controversy as to whether glycogen is converted to lactate to support surrounding neurons or whether glycogen is used glycolytically within astrocytes to spare extracellular glucose for surrounding neurons (Suh et al., 2007; Newman et al., 2011; Suzuki et al., 2011; Brown and Ransom, 2015; Matsui et al., 2017; Duran et al., 2019; Rich et al., 2019; Swanson, 2020). Regardless, increases in extracellular lactate are likely at least partially the result of glycogenolysis. Combined with the glycogen stores, it is also likely that glucose uptake from blood may be able to transiently compensate for the increased demand, thereby resulting in relatively stable extracellular glucose vales.

This work extends previous research and demonstrates that neuronal activation leads to a quick increase in extracellular lactate and more subtle decrease in extracellular glucose within the primary motor cortex of free moving and awake mice engaging in voluntary motor behavior. Additionally, it demonstrates, in awake animals, the additive effect of continuous activation.

### Effort Magnifies Extracellular Metabolite Fluctuations

Previous research has demonstrated that increasing task difficulty increases neuronal demand, which in turn leads to increased cortical activation (Gevins et al., 1997; Winstein et al., 1997; Adler et al., 2001; Bokde et al., 2005; Chen et al., 2008; Siciliano et al., 2017) as well as increased cerebral blood flow (Winstein et al., 1997), oxygen (Causse et al., 2017), glucose uptake (Matsunami et al., 1989; Picard et al., 2013; Sampol et al., 2013; Lundgaard et al., 2015; Díaz-García et al., 2017) and increased extracellular lactate (Urrila et al., 2003; Sampol et al., 2013). We observed an amplification of the metabolite de-coupling during effortful behaviors, when compared to the running behavior. If we consider decreases in extracellular glucose and increases in extracellular lactate as indicators of increased metabolism, effortful behaviors appear to amplify metabolic cortical demand.

There was no additive effect of the spinning behavior on glucose and lactate fluctuations, beyond what was observed in the vertical hold, suggesting that both the spinning and vertical hold conditions similarly engaged the primary motor cortex or that the differences in neural activation across these two conditions did not lie within the primary motor cortex. In muscle, the rate of glycogenolysis can increase with workload (Hearris et al., 2018). Therefore, amplified increases in extracellular lactate produced by effortful behaviors may be the result of increased aerobic glycolysis or elevated rates of glycogenolysis, most likely some combination of the two. Altogether, we propose that the vertical hold paradigm is an appropriate measure of behavior induced neural activation and a useful paradigm for future investigation of awake, freely moving mice, with the advantage over the spinning paradigm that it less likely to result in damaging sensitive equipment.

### Novelty and Experience Differentially Impact Extracellular Fluctuations

In contrast to increases in neuronal activation during challenging tasks, the repetition of a novel or challenging tasks reduces neural activation/firing (Raichle et al., 1994; Gevins et al., 1997; Maccotta and Buckner, 2004; Yamaguchi et al., 2004; van Mulukom et al., 2013; Apšvalka et al., 2015; Satpute et al., 2016) as well as cerebral blood flow (Tsujimoto et al., 2000; Landau et al., 2004; Apšvalka et al., 2015) and metabolic activity (Haier et al., 1992; Picard et al., 2013; Béland-Millar and Messier, 2018). Specifically, Fisher et al. (2016) demonstrated that stereotypic running, following 2 weeks of access to a running wheel, reduced neuronal firing. Zhou et al. (2019) demonstrated that motor learning occurred in two phases. Initial learning and rapid changes in neural activity occurred swiftly after the new behavior, lasting anywhere from a few minutes to a few hours, during which motor errors decreased. Subsequently, substantial cortical reorganization occurred within several weeks of training and was associated with more efficient control of movement and behavior. This bi-phasic response appears to be observed in our results. Long term training/habituation (4 weeks) to a running wheel abolishes the rise in extracellular lactate, suggesting decreased metabolic activity as a result of neuronal efficiency. Short-term habituation (24 h) had the opposite effect, resulting in extracellular lactate levels that surpassed the levels observed on day 1. This contrasted pattern of metabolic activation is similar to what was observed by Sif et al. (1991) during training of a new task. Incomplete training leads to high level of metabolic activity lasting 24 h in relevant brain regions, while mastered training does not (Sif et al., 1991). Similarly, repeated training sessions produce lasting metabolic increases during the first days of training but when the task is mastered, the metabolic activation produced by the trained behavior is limited to less than 5 min (Bontempi, 1996).

This bi-phasic response appears to be restricted to behaviors that have a longer learning curve or require active, rather than passive, participation in learning the behavior. In the novel object task, a relatively passive paradigm, when mice are exposed to a previously novel stimulus for the second time the fluctuations in extracellular metabolites that were observed during the initial presentation are completely absent (Béland-Millar and Messier, 2018). We observed this same trend for behaviors that are not initiated, or controlled, by the mouse (effortful behaviors). During the first exposure (day 1) to the effortful behaviors, the expected de-coupling pattern between extracellular glucose and lactate is observed. This response is significantly altered on day 2. This abolished response is not only different than the trends observed with 24-h of wheel-running exposure (increase in extracellular lactate on day 2) but is similar to the results observed following 4 weeks of exposure to the running wheel.

Beyond novelty and habituation, stress and fitness may also play a role in the observed fluctuations. For the most part, though not controlled, fitness was assumed to be similar given the identical housing and manipulation of the mice. A notable exception, of course, is the group of mice receiving 4-weeks of prior access to the running wheels. On average, these mice ran 14 times further in a day than the mice who just received access to their running wheel, and ran faster (see section “Results”). Alongside the neuronal restructuring that occurs with learning and practice, fitness may have played a role in limiting the rise in extracellular lactate during wheel running with decreased peripheral (blood) lactate production (Favier et al., 1986). Though stress is unlikely to play a significant role in the wheel running data given the habituation and voluntary nature of the behavior, it may have played a role in the testing of the effortful behaviors given the manipulation required to place the mouse on the grid. The impact of stress is challenging to untangle from the impact of the behavior given that both motor behavior and stress are known to increase cerebral lactate (Favier et al., 1986). However, in rats, stressors such as tail pinch and social interactions have resulted in decreased extracellular glucose (Kiyatkin and Lenoir, 2012), which is not observed here.

### Link to Cortical Metabolism

Beyond the ANLS and NALS, one hypothesis focuses on another functional role of lactate production through aerobic glycolysis. It has been suggested that aerobic glycolysis, that is to say glycolysis terminating in the production of lactate rather than pyruvate even in the presence of sufficient oxygen levels, is used to fuel biosynthesis and development and or plasticity of neural networks to support “learning” (Fellows et al., 1993; Bauernfeind et al., 2014; Goyal et al., 2014; Shannon et al., 2016). Intracellular lactate production (or import) alters the redox ratio by increasing intracellular NADH through the equilibrative LDH enzyme. This altered intracellular redox ratio decreases the production of glycolytic intermediates destined for oxidative phosphorylation through negative feedback loops due to the accumulation of various downstream products. This accumulation, in turn, increases the shuttling of the glycolysis/glucose derived carbon through the pentose phosphate pathway. Processing of glucose derived carbon through the pentose phosphate pathway results in the synthesis of various proteins, lipids and nucleotides, necessary for the biosynthesis and development of neural network infrastructure (Raichle, 2015). This is further supported by evidence indicating the need for glycolysis and lactate during memory formation (Suzuki et al., 2011; Steinman et al., 2016; Harris et al., 2019).

The observations presented here are in agreement with this hypothesis, in so much that aerobic glycolysis, and therefore extracellular lactate, would be increased when the behavior is novel as well as when the behavior is being learned (i.e., learning to use the running wheel); and as the behavior becomes “learned” the necessary neural pathways have been built or made more efficient, thus reducing neuronal activation, consequently (according to this hypothesis) diminishing the need for aerobic glycolysis, and therefore extracellular lactate, to support the development of new neural network infrastructure. Indeed, with training, such as in wheel running, vascular GLUT1 expression is increased as well as indicators of neuronal plasticity in the motor cortex and cerebellum (Allen and Messier, 2013). This suggests a strong link between metabolic and cortical reorganization as well as a possible shift to more OXPHOS-oriented metabolism following the development of neural networks and infrastructure. At this juncture, we consider this to be the most likely hypothesis to explain the decrease in extracellular lactate following habituation. However, we cannot discount other hypotheses (e.g., ANLS, NALS) or possibilities such as improved removal or neuronal consumption of lactate, either in neurons or astrocytes. Evidence of increase glucose transporter expression following exercise training could indicate improved glucose oxidation, limiting the need for transfer of lactate, either from glycogen or aerobic glycolysis.

Although our results are not able to distinguish and determine which hypothesis is correct (ANLS, NALS or aerobic glycolysis for biosynthesis), our observed data fit well with the possibility that aerobic glycolysis occurs to fuel biosynthesis. The large increases in extracellular lactate observed during the learning of the wheel running or the first day of spinning would indicate large levels of aerobic glycolysis as the mice engage in and learn new behaviors. Subsequently, the abolished rise in extracellular lactate following training and familiarity would indicate absent, or low levels, of aerobic glycolysis following exposure to these behaviors. Interestingly, the hastened decrease in extracellular glucose following chronic exposure to the running wheel may indicate that extracellular lactate, either blood-borne or as a result of aerobic glycolysis, may have, in part, sustained neuronal metabolic activity.

## Conclusion

This work has extended the observation that neuronal activity results in increases in extracellular lactate and decreases in extracellular glucose within the primary motor cortex. This work has also identified and compared observable voluntary and imposed behaviors as a method of motor cortex activation for use with these sensors. It has demonstrated how these fluctuations can vary with experience, novelty and intensity of motor behaviors. Although our observations do not directly demonstrate the hypothesis, they are nonetheless compatible with the notion that a main function of aerobic glycolysis is not to fuel the current neuronal activity but to sustain new bio-infrastructure as neural networks develop, chiefly through the shuttling of glucose derived carbons into the pentose phosphate pathway for the biosynthesis of nucleotides.

## Data Availability Statement

The raw data supporting the conclusions of this article will be made available by the authors, without undue reservation.

## Ethics Statement

The animal study was reviewed and approved by Animal Care Committee University of Ottawa.

## Author Contributions

AB-M: conceptualization, data curation, formal analysis, writing – original draft, and writing – review and editing. CM: formal analysis, funding acquisition, supervision, writing – original draft, and writing – review and editing. Both authors: contributed to the article and approved the submitted version.

## Conflict of Interest

The authors declare that the research was conducted in the absence of any commercial or financial relationships that could be construed as a potential conflict of interest.

## Publisher’s Note

All claims expressed in this article are solely those of the authors and do not necessarily represent those of their affiliated organizations, or those of the publisher, the editors and the reviewers. Any product that may be evaluated in this article, or claim that may be made by its manufacturer, is not guaranteed or endorsed by the publisher.

## Footnotes

1 Baseline was calculated by averaging 10 s of data prior to each individual event. These 10 s of data were taken 20 s prior to the event to avoid any drift in electrode sensitivity that can occur over hours of testing, and to avoid artificial noise that may occur a few seconds prior to manipulating the mouse (e.g., any accidently jostling of the electrodes when the mouse is picked up).

2 “Practices that reduce data analysis or scientific inference to mechanical “bright-line” rules (such as “*p* < 0.05”) for justifying scientific claims or conclusions can lead to erroneous beliefs and poor decision making. A conclusion does not immediately become “true” on one side of the divide and “false” on the other” (Wasserstein and Lazar, 2016).

## References

[B1] Abdul Muneer P. M. Alikunju S. Szlachetka A. M. Mercer A. J. Haorah J. (2011). Ethanol impairs glucose uptake by human astrocytes and neurons: protective effects of acetyl-L-carnitine. *Int. J. Physiol. Pathophysiol. Pharmacol.* 3 48–56. 21258656 PMC3023411

[B2] Adler C. M. Sax K. W. Holland S. K. Schmithorst V. Rosenberg L. Strakowski S. M. (2001). Changes in neuronal activation with increasing attention demand in healthy volunteers: an fMRI study. *Synapse* 42 266–272. 10.1002/syn.1112 11746725

[B3] Allen A. Messier C. (2013). Plastic changes in the astrocyte GLUT1 glucose transporter and beta-tubulin microtubule protein following voluntary exercise in mice. *Behav. Brain Res.* 240 95–102. 10.1016/j.bbr.2012.11.025 23201358

[B4] Apšvalka D. Gadie A. Clemence M. Mullins P. G. (2015). Event-related dynamics of glutamate and BOLD effects measured using functional magnetic resonance spectroscopy (fMRS) at 3T in a repetition suppression paradigm. *NeuroImage* 118 292–300. 10.1016/j.neuroimage.2015.06.015 26072254

[B5] Bauch E. M. Andreou C. Rausch V. H. Bunzeck N. (2017). Neural habituation to painful stimuli is modulated by dopamine: evidence from a pharmacological fMRI study. *Front. Hum. Neurosci.* 11:630. 10.3389/fnhum.2017.00630 29311880 PMC5742644

[B6] Bauernfeind A. L. Barks S. K. Duka T. Grossman L. I. Hof P. R. Sherwood C. C. (2014). Aerobic glycolysis in the primate brain: reconsidering the implications for growth and maintenance. *Brain Struct. Funct.* 219 1149–1167. 10.1007/s00429-013-0662-z 24185460

[B7] Béland-Millar A. Larcher J. Courtemanche J. Yuan T. Messier C. (2017). Effects of systemic metabolic fuels on glucose and lactate levels in the brain extracellular compartment of the mouse. *Front. Neurosci.* 11:7. 10.3389/fnins.2017.00007 28154523 PMC5243849

[B8] Béland-Millar A. Messier C. (2018). Fluctuations of extracellular glucose and lactate in the mouse primary visual cortex during visual stimulation. *Behav. Brain Res.* 344 91–102. 10.1016/j.bbr.2018.02.018 29458067

[B9] Berg J. Tymoczko J. Stryer L. (2002). *Biochemistry*, 5th Edn. New York: W. H. Freeman.

[B10] Bokde A. L. W. Dong W. Born C. Leinsinger G. Meindl T. Teipel S. J. (2005). Task difficulty in a simultaneous face matching task modulates activity in face fusiform area. *Cogn. Brain Res.* 25 701–710. 10.1016/j.cogbrainres.2005.09.016 16325382

[B11] Bolaños J. P. Almeida A. Moncada S. (2010). Glycolysis: a bioenergetic or a survival pathway? *Trends Biochem. Sci.* 35 145–149. 10.1016/j.tibs.2009.10.006 20006513

[B12] Bontempi B. (1996). Differential temporal evolution of post-training changes in regional brain glucose metabolism induced by repeated spatial discrimination training in mice: visualization of the memory consolidation process? *Eur. J. Neurosci.* 8 2348–2360. 10.1111/j.1460-9568.1996.tb01198.x 8950099

[B13] Brockmann K. (2009). The expanding phenotype of GLUT1-deficiency syndrome. *Brain Dev.* 31 545–552. 10.1016/j.braindev.2009.02.008 19304421

[B14] Brooks G. A. (1985). “Lactate:glycolytic end product and oxidative substrate during sustained exercise in mammals — The “Lactate Shuttle,” in *Circulation, Respiration, and Metabolism: Current Comparative Approaches*, ed. Gilles R. (Berlin: Springer), 208–218. 10.1007/978-3-642-70610-3_15

[B15] Brooks G. A. (1998). Mammalian fuel utilization during sustained exercise. *Comp. Biochem. Physiol. B Biochem. Mol. Biol.* 120 89–107. 10.1016/s0305-0491(98)00025-x 9787780

[B16] Brooks G. A. (2009). Cell-cell and intracellular lactate shuttles. *J. Physiol.* 587 5591–5600. 10.1113/jphysiol.2009.178350 19805739 PMC2805372

[B17] Brown A. M. Ransom B. R. (2015). Astrocyte glycogen as an emergency fuel under conditions of glucose deprivation or intense neural activity. *Metab. Brain Dis.* 30 233–239. 10.1007/s11011-014-9588-2 25037166

[B18] Brunet J. F. Allaman I. Magistretti P. J. Pellerin L. (2010). Glycogen metabolism as a marker of astrocyte differentiation. *J. Cereb. Blood Flow Metab.* 30 51–55. 10.1038/jcbfm.2009.207 19809466 PMC2949090

[B19] Bui T. Thompson C. B. (2006). Cancer’s sweet tooth. *Cancer Cell* 9 419–420. 10.1016/j.ccr.2006.05.012 16766260

[B20] Causse M. Chua Z. Peysakhovich V. Del Campo N. Matton N. (2017). Mental workload and neural efficiency quantified in the prefrontal cortex using fNIRS. *Sci. Rep.* 7:5222. 10.1038/s41598-017-05378-x 28701789 PMC5507990

[B21] Chaigneau E. Tiret P. Lecoq J. Ducros M. Knöpfel T. Charpak S. (2007). The relationship between blood flow and neuronal activity in the rodent olfactory bulb. *J. Neurosci.* 27 6452–6460. 10.1523/JNEUROSCI.3141-06.2007 17567806 PMC6672435

[B22] Chen Y. Martinez-Conde S. Macknik S. L. Bereshpolova Y. Swadlow H. A. Alonso J. M. (2008). Task difficulty modulates the activity of specific neuronal populations in primary visual cortex. *Nat. Neurosci.* 11 974–982. 10.1038/nn.2147 18604204 PMC2553692

[B23] Choeiri C. Staines W. Miki T. Seino S. Messier C. (2005). Glucose transporter plasticity during memory processing. *Neuroscience* 130 591–600. 10.1016/j.neuroscience.2004.09.011 15590143

[B24] Culic O. Gruwel M. L. Schrader J. (1997). Energy turnover of vascular endothelial cells. *Am. J. Physiol.* 273 C205–C213. 10.1152/ajpcell.1997.273.1.C205 9252458

[B25] Di Vito L. Licchetta L. Pippucci T. Baldassari S. Stipa C. Mostacci B. (2018). Phenotype variability of GLUT1 deficiency syndrome: description of a case series with novel SLC2A1 gene mutations. *Epilepsy Behav.* 79 169–173. 10.1016/j.yebeh.2017.12.012 29306089

[B26] Díaz-García C. M. Mongeon R. Lahmann C. Koveal D. Zucker H. Yellen G. (2017). Neuronal stimulation triggers neuronal glycolysis and not lactate uptake. *Cell Metab.* 26 361–374. 10.1016/j.cmet.2017.06.021 28768175 PMC5559896

[B27] Díaz-García C. M. Yellen G. (2018). Neurons rely on glucose rather than astrocytic lactate during stimulation. *J. Neurosci. Res.* 97 883–889. 10.1002/jnr.24374 30575090 PMC6565458

[B28] Dienel G. A. (2012). Brain lactate metabolism: the discoveries and the controversies. *J. Cereb. Blood Flow Metab.* 32 1107–1138. 10.1038/jcbfm.2011.175 22186669 PMC3390802

[B29] Dienel G. A. (2017). Lack of appropriate stoichiometry: strong evidence against an energetically important astrocyte–neuron lactate shuttle in brain. *J. Neurosci. Res.* 95 2103–2125. 10.1002/jnr.24015 28151548

[B30] Dienel G. A. (2018). Brain glucose metabolism: integration of energetics with function. *Physiol. Rev.* 99 949–1045. 10.1152/physrev.00062.2017 30565508

[B31] Dienel G. A. (2019). Does shuttling of glycogen-derived lactate from astrocytes to neurons take place during neurotransmission and memory consolidation? *J. Neurosci. Res.* 97 863–882. 10.1002/jnr.24387 30667077

[B32] Dienel G. A. Cruz N. F. (2015). Contributions of glycogen to astrocytic energetics during brain activation. *Metab. Brain Dis.* 30 281–298. 10.1007/s11011-014-9493-8 24515302 PMC4130810

[B33] Dienel G. A. Hertz L. (2001). Glucose and lactate metabolism during brain activation. *J. Neurosci. Res.* 66 824–838. 10.1002/jnr.10079 11746408

[B34] Dienel G. A. Rothman D. L. (2020). Reevaluation of astrocyte-neuron energy metabolism with astrocyte volume fraction correction: impact on cellular glucose oxidation rates, glutamate-glutamine cycle energetics, glycogen levels and utilization rates vs. exercising muscle, and Na+/K+ pumping rates. *Neurochem. Res.* 45 2607–2630. 10.1007/s11064-020-03125-9 32948935

[B35] DiNuzzo M. Mangia S. Maraviglia B. Giove F. (2010). Glycogenolysis in astrocytes supports blood-borne glucose channeling not glycogen-derived lactate shuttling to neurons: evidence from mathematical modeling. *J. Cereb. Blood Flow Metab.* 30 1895–1904. 10.1038/jcbfm.2010.151 20827264 PMC3002884

[B36] Duran J. Gruart A. Varea O. López-Soldado I. Delgado-García J. M. Guinovart J. J. (2019). Lack of neuronal glycogen impairs memory formation and learning-dependent synaptic plasticity in mice. *Front. Cell. Neurosci.* 13:374. 10.3389/fncel.2019.00374 31456667 PMC6700221

[B37] Eelen G. de Zeeuw P. Treps L. Harjes U. Wong B. W. Carmeliet P. (2018). Endothelial cell metabolism. *Physiol. Rev.* 98 3–58. 10.1152/physrev.00001.2017 29167330 PMC5866357

[B38] Favier R. J. Constable S. H. Chen M. Holloszy J. O. (1986). Endurance exercise training reduces lactate production. *J. Appl. Physiol.* 61 885–889. 10.1152/jappl.1986.61.3.885 3759772

[B39] Fellows L. K. Boutelle M. G. Fillenz M. (1993). Physiological stimulation increases nonoxidative glucose metabolism in the brain of the freely moving rat. *J. Neurochem.* 60 1258–1263. 10.1111/j.1471-4159.1993.tb03285.x 8455025

[B40] Fernández-Moncada I. Ruminot I. Robles-Maldonado D. Alegría K. Deitmer J. W. Barros L. F. (2018). Neuronal control of astrocytic respiration through a variant of the Crabtree effect. *Proc. Natl. Acad. Sci. U. S. A.* 115 1623–1628. 10.1073/pnas.1716469115 29378955 PMC5816174

[B41] Fisher S. P. Cui N. McKillop L. E. Gemignani J. Bannerman D. M. Oliver P. L. (2016). Stereotypic wheel running decreases cortical activity in mice. *Nat. Commun.* 7:13138. 10.1038/ncomms13138 27748455 PMC5071642

[B42] Fox P. T. Raichle M. E. (1986). Focal physiological uncoupling of cerebral blood flow and oxidative metabolism during somatosensory stimulation in human subjects. *Proc. Natl. Acad. Sci. U. S. A.* 83 1140–1144. 10.1073/pnas.83.4.1140 3485282 PMC323027

[B43] Frahm J. Krüger G. Merboldt K. D. Kleinschmidt A. (1996). Dynamic uncoupling and recoupling of perfusion and oxidative metabolism during focal brain activation in man. *Magn. Reson. Med.* 35 143–148. 10.1002/mrm.1910350202 8622575

[B44] Friedman J. R. L. Thiele E. A. Wang D. Levine K. B. Cloherty E. K. Natowicz M. R. (2006). Atypical GLUT1 deficiency with prominent movement disorder responsive to ketogenic diet. *Mov. Disord.* 21 241–245. 10.1002/mds.20660 16149086

[B45] Gevins A. Smith M. E. McEvoy L. Yu D. (1997). High-resolution EEG mapping of cortical activation related to working memory: effects of task difficulty, type of processing, and practice. *Cereb. Cortex* 7 374–385. 10.1093/cercor/7.4.374 9177767

[B46] Ghosh S. Castillo E. Frias E. S. Swanson R. A. (2018). Bioenergetic regulation of microglia. *GLIA* 66 1200–1212. 10.1002/glia.23271 29219210 PMC5903989

[B47] Gonzalez-Lima F. Finkenstädt T. Ewert J. P. (1989). Neural substrates for long-term habituation of the acoustic startle reflex in rats: A 2-deoxyglucose study. *Neurosci. Lett.* 96 151–156. 10.1016/0304-3940(89)90049-9 2927718

[B48] Goyal M. S. Hawrylycz M. Miller J. A. Snyder A. Z. Raichle M. E. (2014). Aerobic glycolysis in the human brain is associated with development and neotenous gene expression. *Cell Metab.* 19 49–57. 10.1016/j.cmet.2013.11.020 24411938 PMC4389678

[B49] Grill-Spector K. Henson R. Martin A. (2006). Repetition and the brain: neural models of stimulus-specific effects. *Trends Cogn. Sci.* 10 14–23. 10.1016/j.tics.2005.11.006 16321563

[B50] Haier R. J. Siegel B. Tang C. Abel L. Buchsbaum M. S. (1992). Intelligence and changes in regional cerebral glucose metabolic rate following learning. *Intelligence* 16 415–426. 10.1016/0160-2896(92)90018-M

[B51] Hall C. N. Klein-Flügge M. C. Howarth C. Attwell D. (2012). Oxidative phosphorylation, not glycolysis, powers presynaptic and postsynaptic mechanisms underlying brain information processing. *J. Neurosci.* 32 8940–8951. 10.1523/JNEUROSCI.0026-12.2012 22745494 PMC3390246

[B52] Harris R. A. Lone A. Lim H. Martinez F. Frame A. K. Scholl T. J. (2019). Aerobic Glycolysis Is Required for Spatial Memory Acquisition But Not Memory Retrieval in Mice. *Eneuro* 6:2019. 10.1523/eneuro.0389-18.2019 30809587 PMC6390195

[B53] Hearris M. A. Hammond K. M. Fell J. M. Morton J. P. (2018). Regulation of muscle glycogen metabolism during exercise: implications for endurance performance and training adaptations. *Nutrients* 10:298. 10.3390/nu10030298 29498691 PMC5872716

[B54] Heilig C. Brosius F. Siu B. Concepcion L. Mortensen R. Heilig K. (2003). Implications of glucose transporter protein type 1 (GLUT1)-haplodeficiency in embryonic stem cells for their survival in response to hypoxic stress. *Am. J. Pathol.* 163 1873–1885. 10.1016/S0002-9440(10)63546-8 14578187 PMC1892427

[B55] Hu Y. Wilson G. S. (1997a). A temporary local energy pool coupled to neuronal activity: fluctuations of extracellular lactate levels in rat brain monitored with rapid-response enzyme-based sensor. *J. Neurochem.* 69 1484–1490. 10.1046/j.1471-4159.1997.69041484.x 9326277

[B56] Hu Y. Wilson G. S. (1997b). Rapid changes in local extracellular rat brain glucose observed with an in vivo glucose sensor. *J. Neurochem.* 68 1745–1752. 10.1046/j.1471-4159.1997.68041745.x 9084449

[B57] Iadecola C. (2017). The neurovascular unit coming of age: a journey through neurovascular coupling in health and disease. *Neuron* 96 17–42. 10.1016/j.neuron.2017.07.030 28957666 PMC5657612

[B58] Ivanov A. I. Malkov A. E. Waseem T. Mukhtarov M. Buldakova S. Gubkina O. (2014). Glycolysis and oxidative phosphorylation in neurons and astrocytes during network activity in hippocampal slices. *J. Cereb. Blood Flow Metab.* 34 397–407. 10.1038/jcbfm.2013.222 24326389 PMC3948126

[B59] Jolivet R. Allaman I. Pellerin L. Magistretti P. J. Weber B. (2010). Comment on recent modeling studies of astrocyte-neuron metabolic interactions. *J. Cereb. Blood Flow Metab.* 30 1982–1986. 10.1038/jcbfm.2010.132 20700131 PMC3002878

[B60] Kass H. R. Winesett S. P. Bessone S. K. Turner Z. Kossoff E. H. (2016). Use of dietary therapies amongst patients with GLUT1 deficiency syndrome. *Seizure* 35 83–87. 10.1016/j.seizure.2016.01.011 26803281

[B61] Kiyatkin E. A. Lenoir M. (2012). Rapid fluctuations in extracellular brain glucose levels induced by natural arousing stimuli and intravenous cocaine: fueling the brain during neural activation. *J. Neurophysiol.* 108 1669–1684. 10.1152/jn.00521.2012 22723672 PMC3544950

[B62] Kiyatkin E. A. Wakabayashi K. T. Lenoir M. (2013). Physiological fluctuations in brain temperature as a factor affecting electrochemical evaluations of extracellular glutamate and glucose in behavioral experiments. *ACS Chem. Neurosci.* 4 652–665. 10.1021/cn300232m 23448428 PMC3656767

[B63] Klepper J. (2012). GLUT1 deficiency syndrome in clinical practice. *Epilepsy Res.* 100 272–277. 10.1016/j.eplepsyres.2011.02.007 21382692

[B64] Klepper J. Diefenbach S. Kohlschütter A. Voit T. (2004). Effects of the ketogenic diet in the glucose transporter 1 deficiency syndrome. *Prostaglandins Leukot. Essent. Fatty Acids* 70 321–327. 10.1016/j.plefa.2003.07.004 14769490

[B65] Klepper J. Leiendecker B. (2013). Glut1 deficiency syndrome and novel ketogenic diets. *J. Child Neurol.* 28 1045–1048. 10.1177/0883073813487600 23666044

[B66] Kozai T. D. Jaquins-Gerstl A. S. Vazquez A. L. Michael A. C. Cui X. T. (2015). Brain tissue responses to neural implants impact signal sensitivity and intervention strategies. *ACS Chem. Neurosci.* 6 48–67. 10.1021/cn500256e 25546652 PMC4304489

[B67] Landau S. M. Schumacher E. H. Garavan H. Druzgal T. J. D’Esposito M. (2004). A functional MRI study of the influence of practice on component processes of working memory. *NeuroImage* 22 211–221. 10.1016/j.neuroimage.2004.01.003 15110011

[B68] Lundgaard I. Li B. Xie L. Kang H. Sanggaard S. Haswell J. D. R. (2015). Direct neuronal glucose uptake heralds activity-dependent increases in cerebral metabolism. *Nat. Commun.* 6 1–12. 10.1038/ncomms7807 25904018 PMC4410436

[B69] Maccotta L. Buckner R. L. (2004). Evidence for neural effects of repetition that directly correlate with behavioral priming. *J. Cogn. Neurosci.* 16 1625–1632. 10.1162/0898929042568451 15601524

[B70] Madsen P. L. Hasselbalch S. G. Hagemann L. P. Olsen K. S. Bülow J. Holm S. (1995). Persistent resetting of the cerebral oxygen/glucose uptake ratio by brain activation: evidence obtained with the Kety-Schmidt technique. *J. Cereb. Blood Flow Metab.* 15 485–491. 10.1038/jcbfm.1995.60 7714007

[B71] Magistretti P. J. (2006). Neuron-glia metabolic coupling and plasticity. *J. Exp. Biol.* 209 2304–2311. 10.1242/jeb.02208 16731806

[B72] Magistretti P. J. Allaman I. (2015). A Cellular Perspective on Brain Energy Metabolism and Functional Imaging. *Neuron* 86 883–901. 10.1016/j.neuron.2015.03.035 25996133

[B73] Magistretti P. J. Allaman I. (2018). Lactate in the brain: from metabolic end-product to signalling molecule. *Nat. Rev. Neurosci.* 19 235–249. 10.1038/nrn.2018.19 29515192

[B74] Mangia S. Dinuzzo M. Giove F. Carruthers A. Simpson I. A. Vannucci S. J. (2011). Response to comment on recent modeling studies of astrocyte-neuron metabolic interactions: much ado about nothing. *J. Cereb. Blood Flow Metab.* 31 1346–1353. 10.1038/jcbfm.2011.29 21427731 PMC3130323

[B75] Marin-Valencia I. Good L. B. Ma Q. Duarte J. Bottiglieri T. Sinton C. M. (2012). Glut1 deficiency (G1D): epilepsy and metabolic dysfunction in a mouse model of the most common human phenotype. *Neurobiol. Dis.* 48 92–101. 10.1016/j.nbd.2012.04.011 22683290 PMC3495165

[B76] Mason S. (2017). Lactate shuttles in neuroenergetics-homeostasis, allostasis and beyond. *Front. Neurosci.* 11:43. 10.3389/fnins.2017.00043 28210209 PMC5288365

[B77] Matsui T. Omuro H. Liu Y. F. Soya M. Shima T. McEwen B. S. (2017). Astrocytic glycogen-derived lactate fuels the brain during exhaustive exercise to maintain endurance capacity. *Proc. Natl. Acad. Sci. U. S. A.* 114 6358–6363. 10.1073/pnas.1702739114 28515312 PMC5474817

[B78] Matsunami K. Kawashima T. Satake H. (1989). Mode of [14C] 2-deoxy-d-glucose uptake into retrosplenial cortex and other memory-related structures of the monkey during a delayed response. *Brain Res. Bull.* 22 829–838. 10.1016/0361-9230(89)90026-9 2765943

[B79] McNay E. C. Fries T. M. Gold P. E. (2000). Decreases in rat extracellular hippocampal glucose concentration associated with cognitive demand during a spatial task. *Proc. Natl. Acad. Sci. U. S. A.* 97 2881–2885. 10.1073/pnas.050583697 10706633 PMC16024

[B80] McNay E. C. Gold P. E. (2001). Age-related differences in hippocampal extracellular fluid glucose concentration during behavioral testing and following systemic glucose administration. *J. Gerontol. A Biol. Sci. Med. Sci.* 56 B66–B71. 10.1093/gerona/56.2.B66 11213269

[B81] McNay E. C. McCarty R. C. Gold P. E. (2001). Fluctuations in brain glucose concentration during behavioral testing: dissociations between brain areas and between brain and blood. *Neurobiol. Learn. Mem.* 75 325–337. 10.1006/nlme.2000.3976 11300738

[B82] McNay E. C. Sherwin R. S. (2004). Effect of recurrent hypoglycemia on spatial cognition and cognitive metabolism in normal and diabetic rats. *Diabetes* 53 418–425. 10.2337/diabetes.53.2.418 14747293

[B83] McNay E. C. Williamson A. McCrimmon R. J. Sherwin R. S. (2006). Cognitive and neural hippocampal effects of long-term moderate recurrent hypoglycemia. *Diabetes* 55 1088–1095. 10.2337/diabetes.55.04.06.db05-1314 16567533

[B84] Mergenthaler P. Lindauer U. Dienel G. A. Meisel A. (2013). Sugar for the brain: the role of glucose in physiological and pathological brain function. *Trends Neurosci.* 36 587–597. 10.1016/j.tins.2013.07.001 23968694 PMC3900881

[B85] Mintun M. A. Lundstrom B. N. Snyder A. Z. Vlassenko A. G. Shulman G. L. Raichle M. E. (2001). Blood flow and oxygen delivery to human brain during functional activity: theoretical modeling and experimental data. *Proc. Natl. Acad. Sci. U. S. A.* 98 6859–6864. 10.1073/pnas.111164398 11381119 PMC34443

[B86] Newman L. A. Korol D. L. Gold P. E. (2011). Lactate produced by glycogenolysis in astrocytes regulates memory processing. *PLoS One* 6:e28427. 10.1371/journal.pone.0028427 22180782 PMC3236748

[B87] Patching S. G. (2017). Glucose transporters at the blood-brain barrier: function, regulation and gateways for drug delivery. *Mol. Neurobiol.* 54 1046–1077. 10.1007/s12035-015-9672-6 26801191

[B88] Paulson O. B. Hasselbalch S. G. Rostrup E. Knudsen G. M. Pelligrino D. (2010). Cerebral blood flow response to functional activation. *J. Cereb. Blood Flow Metab.* 30 2–14. 10.1038/jcbfm.2009.188 19738630 PMC2872188

[B89] Paxinos G. Franklin K. B. J. (2008). *The Mouse Brain In Stereotaxic Coordinates* - Third Edition. Cambridge: Academic Press, 10.1016/S0306-4530(03)00088-X

[B90] Pellerin L. Magistretti P. J. (1994). Glutamate uptake into astrocytes stimulates aerobic glycolysis: a mechanism coupling neuronal activity to glucose utilization. *Proc. Natl. Acad. Sci. U. S. A.* 91 10625–10629. 10.1073/pnas.91.22.10625 7938003 PMC45074

[B91] Pellerin L. Magistretti P. J. (2012). Sweet sixteen for ANLS. *J. Cereb. Blood Flow Metab.* 32 1152–1166. 10.1038/jcbfm.2011.149 22027938 PMC3390819

[B92] Picard N. Matsuzaka Y. Strick P. L. (2013). Extended practice of a motor skill is associated with reduced metabolic activity in M1. *Nat. Neurosci.* 16 1340–1347. 10.1038/nn.3477 23912947 PMC3757119

[B93] Raichle M. E. (2015). The restless brain: how intrinsic activity organizes brain function. *Philos. Trans. R. Soc. B Biol. Sci.* 370:20140172. 10.1098/rstb.2014.0172 25823869 PMC4387513

[B94] Raichle M. E. Fiez J. A. Videen T. O. Macleod A. Mary K. Pardo J. V. (1994). Practice-related changes in human brain functional anatomy during nonmotor learning. *Cereb. Cortex* 4 8–26. 10.1093/cercor/4.1.8 8180494

[B95] Rangaraju V. Calloway N. Ryan T. A. (2014). Activity-driven local ATP synthesis is required for synaptic function. *Cell* 156 825–835. 10.1016/j.cell.2013.12.042 24529383 PMC3955179

[B96] Rich L. R. Harris W. Brown A. M. (2019). The role of brain glycogen in supporting physiological function. *Front. Neurosci.* 13:1176. 10.3389/fnins.2019.01176 31749677 PMC6842925

[B97] Sampol D. Ostrofet E. Jobin M. L. Raffard G. Sanchez S. Bouchaud V. (2013). Glucose and lactate metabolism in the awake and stimulated rat: a 13C-NMR study. *Front. Neuroenerg.* 5:5. 10.3389/fnene.2013.00005 23755012 PMC3668265

[B98] Satpute A. B. Hanington L. Barrett L. F. (2016). Novel response patterns during repeated presentation of affective and neutral stimuli. *Soc. Cogn. Affect. Neurosci.* 11 1919–1932. 10.1093/scan/nsw104 27928070 PMC5141956

[B99] Scavuzzo C. J. Newman L. A. Gold P. E. Korol D. L. (2020). Extracellular levels of glucose in the hippocampus and striatum during maze training for food or water reward in rats. *BioRxiv* [Preprint]. 10.1101/2020.04.20.051284 PMC8238909 34048874

[B100] Shannon B. J. Vaishnavi S. N. Vlassenko A. G. Shimony J. S. Rutlin J. Raichle M. E. (2016). Brain aerobic glycolysis and motor adaptation learning. *Proc. Natl. Acad. Sci. U. S. A.* 113 E3782–E3791. 10.1073/pnas.1604977113 27217563 PMC4932971

[B101] Siciliano R. E. Madden D. J. Tallman C. W. Boylan M. A. Kirste I. Monge Z. A. (2017). Task difficulty modulates brain activation in the emotional oddball task. *Brain Res.* 1664 74–86. 10.1016/j.brainres.2017.03.028 28377158 PMC5452685

[B102] Sif J. Messier C. Meunier M. Bontempi B. Calas A. Destrade C. (1991). Time-dependent sequential increases in [14C]2-deoxyglucose uptake in subcortical and cortical structures during memory consolidation of an operant training in mice. *Behav. Neural Biol.* 56 43–61. 10.1016/0163-1047(91)90279-Y 1867626

[B103] Simpson I. A. Carruthers A. Vannucci S. J. (2007). Supply and demand in cerebral energy metabolism: the role of nutrient transporters. *J. Cereb. Blood Flow Metab.* 27 1766–1791. 10.1038/sj.jcbfm.9600521 17579656 PMC2094104

[B104] Steinman M. Q. Gao V. Alberini C. M. (2016). The Role of Lactate-Mediated Metabolic Coupling between Astrocytes and Neurons in Long-Term Memory Formation. *Front. Integr. Neurosci.* 10:10. 10.3389/fnint.2016.00010 26973477 PMC4776217

[B105] Suh S. W. Bergher J. P. Anderson C. M. Treadway J. L. Fosgerau K. Swanson R. A. (2007). Astrocyte glycogen sustains neuronal activity during hypoglycemia: studies with the glycogen phosphorylase inhibitor CP-316,819 ([R-R*,S*]-5-chloro-N-[2-hydroxy-3-(methoxymethylamino)-3-oxo-1-(phenylmethyl)propyl]-1H-indole-2-carboxamide). *J. Pharmacol. Exp. Ther.* 321 45–50. 10.1124/jpet.106.115550 17251391

[B106] Suzuki A. Stern S. A. Bozdagi O. Huntley G. W. Walker R. H. Magistretti P. J. (2011). Astrocyte-neuron lactate transport is required for long-term memory formation. *Cell* 144 810–823. 10.1016/j.cell.2011.02.018 21376239 PMC3073831

[B107] Swanson R. A. (2020). A thermodynamic function of glycogen in brain and muscle. *Progr. Neurobiol.* 189:101787. 10.1016/j.pneurobio.2020.101787 32151532 PMC11156230

[B108] Takita M. Mikuni M. Takahashi K. (1992). Habituation of lactate release responding to stressful stimuli in rat prefrontal cortex in vivo. *Am. J. Physiol. Regul. Integr. Comp. Physiol.* 263 R722–R727. 10.1152/ajpregu.1992.263.3.r722 1415663

[B109] Toga A. W. Collins R. C. (1981). Glucose metabolism increases in visual pathways following habituation. *Physiol. Behav.* 27 825–834. 10.1016/0031-9384(81)90049-4 7323190

[B110] Tsujimoto T. Ogawa M. Tsukada H. Kakiuchi T. Sasaki K. (2000). Decline of the monkey’s limbic and prefrontal activity during task repetition. *Neurosci. Lett.* 283 69–72. 10.1016/S0304-3940(00)00913-7 10729636

[B111] Urrila A. S. Hakkarainen A. Heikkinen S. Vuori K. Stenberg D. Häkkinen A. M. (2003). Metabolic imaging of human cognition: an fMRI/1H-MRS study of brain lactate response to silent word generation. *J. Cereb. Blood Flow Metab.* 23 942–948. 10.1097/01.WCB.0000080652.64357.1D 12902838

[B112] van Mulukom V. Schacter D. L. Corballis M. C. Addis D. R. (2013). Re-Imagining the future: repetition decreases hippocampal involvement in future simulation. *PLoS One* 8:e69596. 10.1371/journal.pone.0069596 23936055 PMC3720617

[B113] Wakabayashi K. T. Kiyatkin E. A. (2012). Rapid changes in extracellular glutamate induced by natural arousing stimuli and intravenous cocaine in the nucleus accumbens shell and core. *J. Neurophysiol.* 108 285–299. 10.1152/jn.01167.2011 22496525 PMC3434613

[B114] Wakabayashi K. T. Kiyatkin E. A. (2015). Central and peripheral contributions to dynamic changes in nucleus accumbens glucose induced by intravenous cocaine. *Front. Neurosci.* 9:42. 10.3389/fnins.2015.00042 25729349 PMC4325903

[B115] Wang Y. Wang R. Xu X. (2017). Neural energy supply-consumption properties based on hodgkin-huxley model. *Neural Plast.* 2017:6207141. 10.1155/2017/6207141 28316842 PMC5337805

[B116] Wasserstein R. Lazar N. (2016). The ASA’s Statement on p-Values: context, process, and purpose. *Am. Stat.* 70 129–133. 10.1080/00031305.2016.1154108

[B117] Watts M. E. Pocock R. Claudianos C. (2018). Brain energy and oxygen metabolism: emerging role in normal function and disease. *Front. Mol. Neurosci.* 11:216. 10.3389/fnmol.2018.00216 29988368 PMC6023993

[B118] Wilson G. S. Gifford R. (2005). Biosensors for real-time in vivo measurements. *Biosens Bioelect.* 20 2388–2403. 10.1016/j.bios.2004.12.003 15854814

[B119] Winstein C. J. Grafton S. T. Pohl P. S. (1997). Motor task difficulty and brain activity: investigation of goal- directed reciprocal aiming using positron emission tomography. *J. Neurophysiol.* 77 1581–1594. 10.1152/jn.1997.77.3.1581 9084621

[B120] Yamaguchi S. Hale L. A. D’Esposito M. Knight R. T. (2004). Rapid prefrontal-hippocampal habituation to novel events. *J. Neurosci.* 24 5356–5363. 10.1523/JNEUROSCI.4587-03.2004 15190108 PMC6729309

[B121] Yellen G. (2018). Fueling thought: management of glycolysis and oxidative phosphorylation in neuronal metabolism. *J. Cell Biol.* 217 2235–2246. 10.1083/jcb.201803152 29752396 PMC6028533

[B122] Yetkin-Arik B. Vogels I. M. C. Nowak-Sliwinska P. Weiss A. Houtkooper R. H. Van Noorden C. J. F. (2019). The role of glycolysis and mitochondrial respiration in the formation and functioning of endothelial tip cells during angiogenesis. *Sci. Rep.* 9:12608. 10.1038/s41598-019-48676-2 31471554 PMC6717205

[B123] Yu M. Sun N. Buajieerguli M. Xie Y. Meng H. (2020). The glucose transporter type 1 deficiency syndrome. *Chin. J. Neurol.* 53 138–142. 10.3760/cma.j.issn.1006-7876.2020.02.012 30704229

[B124] Zhou X. Tien R. N. Ravikumar S. Chase S. M. (2019). Distinct types of neural reorganization during long-term learning. *J. Neurophysiol.* 121 1329–1341. 10.1152/jn.00466.2018 30726164 PMC6485743

